# Identifying *AIM2* Circulating Methylation Levels as a Novel Diagnostic Biomarker for Rheumatoid Arthritis Using Targeted DNA Methylation Sequencing

**DOI:** 10.2174/0118715303401357250707080740

**Published:** 2025-07-17

**Authors:** Jianan Zhao, Binghen He, Yu Shan, Kai Wei, Ping Jiang, Yiming Shi, Cen Chang, Yixin Zheng, Fuyu Zhao, Yunshen Li, Yuejuan Zheng, Yehua Jin, Xinliang Lv, Mengru Guo

**Affiliations:** 1 Department of Rheumatology, Shanghai Guanghua Hospital of Integrative Medicine, Shanghai University of Traditional Chinese Medicine, Shanghai, China;; 2 Guanghua Clinical Medical College, Shanghai University of Traditional Chinese Medicine, Shanghai, China;; 3 Institute of Arthritis Research in Integrative Medicine, Shanghai Academy of Traditional Chinese Medicine, Shanghai, China;; 4 Shuguang Clinical Medical College, Shanghai University of Traditional Chinese Medicine Affiliated Shuguang Hospital, Shanghai, China;; 5 The Research Center for Traditional Chinese Medicine, Shanghai Institute of Infectious Diseases and Biosecurity, Shanghai University of Traditional Chinese Medicine, Shanghai, China;; 6 Inner Mongolia Autonomous Region Hospital of Traditional Chinese Medicine, Hohhot, Inner Mongolia Autonomous Region, China

**Keywords:** Rheumatoid arthritis, DNA methylation, *AIM2* protein, anti-TNF-α treatment, diabetes, epigenesis, genetic

## Abstract

**Introduction:**

This study investigated the association between *AIM2* cg11003133 DNA methylation and Rheumatoid Arthritis (RA), evaluating its diagnostic potential for RA and subtypes.

**Methods:**

MethylTarget™ sequencing targeted *AIM2* cg11003133 (chr1:159076528-159076740) in RA, Ankylosing Spondylitis (AS), Psoriatic Arthritis (PsA), gout, Systemic Lupus Erythematosus (SLE), Dermatomyositis (DM), primary Sjögren's Syndrome (SS), and Healthy Controls (HC) patients. Logistic regression, random forest, and XGBoost models were applied, with Spearman’s correlation used to assess associations.

**Results:**

RA and RF/CCP-positive patients showed significantly higher methylation at cg11003133_79/91 compared to HC and AS (*FDR* < 0.05), but lower levels compared to DM. Methylation at cg11003133_139 was elevated in RA compared to AS/SS (*FDR* = 0.04/0.03). Anti-TNF-α non-responders had higher cg11003133_79/91 methylation levels compared to HC/AS non-responders (*FDR* < 0.05). RF-negative RA patients had higher cg11003133_91 methylation than AS patients who failed anti-TNF-α treatment (*FDR* < 0.05). Haplotype CCCC correlated positively with CRP (r = 0.14, *P* = 0.006); TTTT was significantly negatively correlated with erythrocyte sedimentation rate, CRP, and the presence of diabetes (r = -0.18, -0.15, and -0.14; *P* < 0.001, 0.003, and 0.008, respectively). XGBoost and RF models achieved AUCs of 0.9911 and 0.9975 for RA *versus* non-RA, and 1 for RF/CCP double-negative *versus* double-positive RA.

**Discussion:**

*AIM2* cg11003133 methylation is strongly linked to RA, aligning with its role in inflammasome activation. While promising for diagnostics, larger validation is needed.

**Conclusions:**

*AIM2* cg11003133 methylation may serve as a diagnostic biomarker for RA and subtypes.

## 1 INTRODUCTION

Rheumatoid Arthritis (RA) is a chronic autoimmune disease affecting multiple organs and systems, characterized by chronic synovitis, the presence of autoantibodies, and persistent joint and bone damage. The aetiology of RA is complex and heterogeneous [1], and its development is associated with numerous factors, including genetics, metabolism, and immunology. Among these, genetics plays a crucial role, with a particular focus on genetic predisposition, such as the Major Histocompatibility Complex (MHC) molecules, which are critical immune-related genes associated with RA susceptibility. In addition to genetic predisposition, research has increasingly emphasized the role of epigenetic modifications, which are distinct from traditional genetic factors, in influencing RA pathogenesis [2]. RA is associated with various autoantibodies, including Rheumatoid Factor (RF) and Cyclic Citrullinated Peptides (CCP), which are important diagnostic markers. They are crucial for diagnosing seropositive patients; approximately one-third of RA patients are seronegative, complicating early diagnosis and intervention, which are key challenges in RA management. Delayed or inaccurate diagnosis can cause irreversible joint damage and a rapid decline in the quality of life. Therefore, early diagnosis and intervention are crucial for disease management, significantly improving clinical outcomes and reducing disability rates.

Absent In Melanoma 2 (*AIM2*), a crucial Pattern Recognition Receptor (PRR), detects viral and bacterial DNA. In RA, accumulating evidence suggests that extracellular DNA may contribute to disease onset and progression, potentially influencing *AIM2* expression. For instance, in mouse models deficient in DNase II, defects in DNA processing lead to ectopic localisation and accumulation in the cytoplasm, which activates STING, thereby enhancing the production of type I interferons (IFNs) and pro-inflammatory cytokines, eventually leading to the development of a polyarthritis model [3]. *AIM2* can act through inflammasome-dependent and -independent pathways in several RA effector cells, which are primarily related to inflammatory and immune responses [4]. High expression of *AIM2* in RA Fibroblast-Like Synoviocytes (FLS) may promote abnormal invasion, cell proliferation, and the release of inflammatory factors, whereas *AIM2* siRNA can inhibit this process [5]. Phosphoglycerate mutase family member 5 interacts with *AIM2* to enhance *AIM2* inflammasome assembly and inflammation in macrophages [6]. *AIM2* may also affect RA by influencing T-cell subset development [4]. These findings suggest that *AIM2* is intricately linked to the inflammatory and immune dysregulation observed in RA, making it a promising candidate for further investigation.

DNA methylation, a key aspect of epigenetic research, is increasingly studied about RA. Whole-genome analyses of monozygotic twins discordant for RA demonstrate increased variability in DNA methylation, suggesting that epigenetic modifications may be associated with autoimmune disease pathogenesis [7]. DNA methylation can potentially influence pathogenic mechanisms of RA by modulating various effector cells involved in the disease, such as those affecting T cell differentiation, and inflammatory and immune responses [8]. For example, methylation of phosphatase and tensin homologue (*PTEN*) has been associated with the promotion of inflammatory responses and activation of an invasive phenotype in synovial fibroblasts in animal models of experimental arthritis [9]. Similarly, high DNA methylation of *PTCH1* is associated with the persistent activation of synovial fibroblasts and inflammation. Reducing the DNA methylation levels of *PTCH1* may inhibit the activation of the hedgehog signalling pathway, potentially mitigating inflammation and improving RA [9]. Additionally, differential DNA methylation sites have been identified in peripheral blood mononuclear cells from RA patients, which may correlate with various effects on effector cells, including cell proliferation, cell cycle regulation, cell viability, and the expression of inflammatory cytokines [10]. Gene expression and Treg-specific hypomethylation of Forkhead box protein 3 (*FOXP3*) are significantly reduced, which may play a role in RA pathogenesis [11]. Furthermore, several studies have highlighted the link between differential DNA methylation regions and diagnostic/therapeutic advancements in RA. Specific DNA methylation regions in T cells have been suggested as potential diagnostic biomarkers for RA [8]. RA patients exhibit cell-specific differential methylation patterns before and after Methotrexate (MTX) treatment, which may correlate with treatment response variability [12]. Anti-CCP-positive RA patients show significant differential methylation in their peripheral blood mononuclear cells compared to anti-CCP-negative patients [13]. Additionally, DNA methylation in homeodomain interacting protein kinase 3, C-X-C motif chemokine receptor 5, and 5-hydroxytryptamine receptor 2A in peripheral blood mononuclear cells may serve as potential diagnostic markers for RA [14-16].

Given the established role of *AIM2* in RA pathogenesis and the potential of DNA methylation as a diagnostic tool, this study aimed to investigate the association between DNA methylation at *AIM2* cg11003133 and RA. Notably, while RA is the primary focus of this study, other autoimmune rheumatic diseases—Ankylosing Spondylitis (AS), Psoriatic Arthritis (PsA), gout, Systemic Lupus Erythematosus (SLE), Dermatomyositis (DM), and primary Sjögren's syndrome (SS) are also included—to evaluate whether methylation patterns at *AIM2* are specific to RA or shared among related conditions. These diseases were selected based on their overlapping clinical features and shared pathogenic mechanisms with RA, such as chronic inflammation, immune dysregulation, and genetic predisposition. By including these diseases, the aim is to provide a broader context for understanding the specificity and potential diagnostic utility of *AIM2* methylation in RA. Building on previous findings from our group, which identified *AIM2* cg11003133_7 as a candidate site through multiomics analysis (unpublished), targeted DNA methylation sequencing was employed to validate and extend these observations. This study aims not only to elucidate the role of *AIM2* methylation in RA pathogenesis but also explores its potential as a diagnostic biomarker for RA and its subtypes, addressing the challenges of seronegative RA and treatment response variability.

## 2 MATERIALS AND METHODS

### 2.1 Participants and Peripheral Blood Collection

Patient recruitment was conducted within the Guanghua Hospital Precision Medicine Research Cohort (PMRC) at the Shanghai University of Traditional Chinese Medicine. The cohort included 166 patients with various conditions: Rheumatoid Arthritis (RA), Ankylosing Spondylitis (AS), Psoriatic Arthritis (PsA), gout, Systemic Lupus Erythematosus (SLE), Dermatomyositis (DM), and Healthy Controls (HC); with 30 individuals per group, except for 24 patients with primary Sjögren’s Syndrome (SS). RA patients were further categorized into four serological subtypes: RA_DP (positive for both Rheumatoid Factor (RF) and anti-Cyclic Citrullinated Peptide (CCP) antibodies), RA_DN (negative for both RF and CCP), RA_RFN (RF-negative), and RA_CCPN (CCP-negative). Additionally, RA patients were divided based on their clinical response to anti-TNF-alpha therapy into responders (RA_AJN_Y, defined as achieving a DAS28 improvement >1.2, regardless of whether DAS28 was calculated using ESR or CRP) and non-responders (RA_AJN_N, defined as DAS28 improvement ≤ 1.2). Similarly, AS patients were categorized based on their response to anti-TNF-alpha therapy into responders (AS_Y, defined as achieving a post-treatment ASDAS < 1.3) and non-responders (AS_N, failing to meet this threshold). Inclusion criteria for each disease followed the respective classification standards: the 2010 American College of Rheumatology (ACR) classification criteria for RA [17], the modified 1984 New York criteria for AS [18], the 2015 ACR/European Alliance of Associations for Rheumatology (EULAR) classification criteria for gout [19], the 2006 ACR classification criteria for PsA [20], the 2019 EULAR/ACR classification criteria for SLE [21], the 2017 ACR/EULAR classification criteria for DM [22], and the 2016 ACR/EULAR classification criteria for primary SS [23]. Patients with concurrent autoimmune diseases, severe hepatic or renal impairment, cardiovascular diseases, or a history of malignancies were exclude. Complete clinical information was thoroughly documented for all individuals, and whole blood samples were collected (Table **1**). Informed consent was obtained from all participants, and ethical approval was granted by the Ethics Committee of Guanghua Hospital (Approval No.: 2023-K-32).

### 2.2 Targeted DNA Methylation Analysis

Targeted DNA methylation analysis involves sample quality control, PCR primer design and optimisation, bisulfite treatment, PCR amplification with specific labels, and high-throughput sequencing. Genomic DNA is extracted from the peripheral blood of recruited patients and subject to quality control, with required standards of a concentration ≥20 ng/μL and a total amount of ≥400 ng. Purity criteria include OD260/280 = 1.7~1.9; OD260/230 ≥ 2.0. Primers are designed and optimised using the “Methylation FastTarget V4.1” software, with the forward primer (PrimerF) sequence as *GAAAGTTATTTAGGTTATTTGGGTATGTT* and the reverse primer (PrimerR) sequence as *TTCTCAAAAATATACACAAC*, targeting the cg site cg11003133_7. The sequence length spans 213bp from chr1:159076740 to chr1:159076528. Subsequently, the samples were subjected to multiplex PCR amplification aimed at specific fragments, followed by high-throughput sequencing on the Illumina HiSeq (Illumina, CA, USA) platform in paired-end sequencing mode with 2 × 150 *bp* read lengths, resulting in FastQ data. Primer design and optimisation were performed using “Methylation FastTarget V4.1” software. The acquired FastQ data then underwent upstream data processing, which primarily included quality assessment, merging of R1/R2 reads, alignment and filtering against a reference sequence, counting the number of valid reads, and secondary alignment against the reference sequence. Finally, a methylation expression matrix was obtained for subsequent downstream analysis. The methylation level of CpG sites within the amplicons was calculated as follows: the methylation level = the number of reads showing methylation at a given CpG site (*i.e*., reads detecting cytosine [C]) / the total number of reads covering the CpG site. Haplotype analysis of CpG site methylation was performed at the amplicon level. For example, assuming the amplicon sequence is “*ATCATXGATCXGCTAXGCTTTAXGCCTAT,*” where “*X*” can represent “*C*” (methylated) or “*T*” (unmethylated). If one sequencing read is “*ATCATCGATCTGCTACGCTTTATGCCTAT*,” then the methylation haplotype of the amplicon corresponding to this read is “*CTCT*.” For each sample, the proportion of a specific methylation haplotype was calculated as the number of reads for that haplotype divided by the total number of reads for the amplicon. The specific sequencing depth information is provided in Table **S1**.

### 2.3 Statistical Methods

Statistical analyses were performed using R software (Version 4.2; R Foundation for Statistical Computing, Vienna, Austria) for logistic regression, random forest, and XGBoost modelling, as well as visualisation and statistical analysis. Various packages were employed, including “patchwork”, “ggplot2”, “readxl”, “tidyverse”, “reshape2”, “ggrepel”, “mice”, “Hmisc”, “pheatmap”, “ggtree”, “aplot”, “tidyr”, “magrittr”, “ggcor”, “ggpubr”, “ggthemes”, “caret”, “pROC”, “MatchIt”, “shapviz”, “xgboost”, “ROCit”, and “randomForest”. Random forest and XGBoost modelling used 5-fold cross-validation and randomly split the dataset into training and testing sets in a 7:3 ratio. The XGBoost analysis was implemented using the xgboost and caret packages in R. The matched dataset was split into 70% training and 30% testing subsets. Initial training parameters included objective = “binary:logistic”, booster = “gbtree”, eval_metric = “error”, eta = 0.3, max_depth = 3, subsample = 1, colsample_bytree = 1, and gamma = 0.5. Hyperparameter tuning was performed using grid search with 5-fold cross-validation over a predefined grid of values for eta, max_depth, gamma, subsample, and nrounds. The optimal parameters selected through this process were eta = 0.1, max_depth = 3, gamma = 0.25, subsample = 0.5, and 100 boosting rounds. The final model was then trained using the optimised parameters and evaluated on the testing dataset. Model performance metrics, including the Area Under the ROC Curve (AUC), R^2^, Root Mean Square Error (RMSE), and Mean Absolute Error (MAE), were calculated. Missing data were handled using multiple imputations *via* the “mice” package in R. This approach uses chained equations to estimate missing values based on available data, ensuring that the imputed values reflect the underlying data distribution. The imputation process was conducted to maintain data completeness and minimise the bias introduced by missing values. Comparability was ensured through propensity score matching. The Spearman's method (ρ) was used for correlation analysis and visualisation. To address multiple testing, all correlation *p*- values underwent Benjamini-Hochberg False Discovery Rate (FDR) correction, with *q* < 0.05 considered significant. Correlation coefficients were interpreted as: |ρ| < 0.20 (negligible), 0.20-0.39 (weak), ≥0.40 (moderate/strong).

Data following a normal distribution were expressed as mean ± standard deviation, with comparisons among multiple groups conducted using Analysis of Variance (ANOVA). Data not following a normal distribution were presented as median (Q1, Q3); comparisons among multiple groups were performed using the Kruskal-Wallis method. For significant omnibus Kruskal-Wallis results (FDR-adjusted *q* < 0.05), Dunn’s tests with FDR correction were applied for pairwise comparisons. All reported *p*-values from post-hoc analyses reflect FDR-adjusted *q*-values.

## 3 RESULTS

### 3.1 Methylation Levels of *AIM2* Significantly Differ Between RA and Other Diseases

**Fig. (1):**
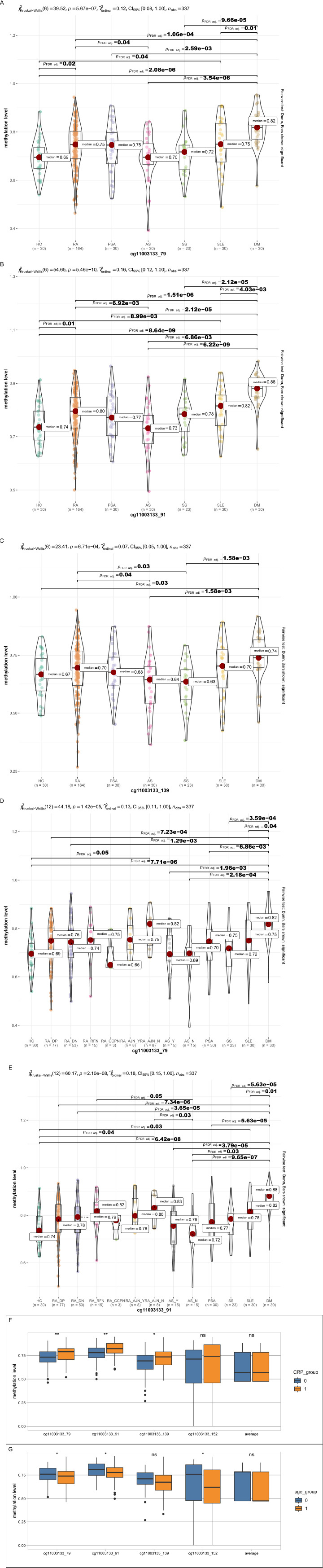
Methylation levels at the *AIM2* cg11003133. (**A-E**) Multiple comparisons of methylation levels at different CG sites of *AIM2* cg11003133 across various groups, with FDR <0.05 indicating statistically significant. (**F-G**) Methylation levels at different CG sites of *AIM2* cg11003133 in RA patients under different clinical cut-off values, with *P* <0.05 being statistically significant.

Targeted methylation analysis of the *AIM2* region, spanning 213 *bp* from chr1:159076740 to chr1:159076528, identified four CpG sites: cg11003133_79(chr1:159076662), cg11003133_91(chr1:159076650), cg11003133_139(chr1: 159076602), and cg11003133_152(chr1:159076589). Statistical analysis revealed that methylation levels at these sites significantly differed across disease groups, highlighting the potential relevance of *AIM2* methylation as a biomarker for differentiating RA from other conditions. Specifically, cg11003133_79 methylation was significantly higher in RA patients compared to the HC and AS groups (*FDR* = 0.02 and 0.04, respectively), but lower compared to the DM group (*FDR* = 1.06 x 10^-4^). Similarly, cg11003133_91 showed a higher methylation level in RA patients compared to the HC and AS groups (*FDR* = 0.01 and 6.92 x 10^-3^, respectively) and a lower methylation level compared to the DM group (*FDR* = 1.51 x 10^-6^). Additionally, cg11003133_139 methylation levels were higher in RA patients compared to the AS and SS groups (FDR = 0.04 and 0.03, respectively) (Figs. **1A**-**C**).

To further investigate the clinical relevance of methylation changes, differences between RA subtypes and their association with treatment response and serological indicators were analyzed. Methylation at cg11003133_79 was significantly lower in both RF/CCP double-positive and double-negative RA patients than in the DM group (*FDR* = 7.5 × 10^-4^ and 1.5 × 10^-3^, respectively). Interestingly, RA patients who were non-responsive to anti-TNF-α treatment exhibited significantly higher levels of cg11003133_79 methylation compared to the HC group (*FDR* = 0.05). Similarly, cg11003133_91 methylation levels were lower in RF/CCP double-positive and double-negative patients compared to the DM group (*FDR* = 7.34 × 10^^-6^ and 3.65 × 10^^-5^, respectively). In comparison, RF single-negative patients exhibited significantly higher levels compared to non-responsive AS patients (*FDR* = 0.05). Non-responsive RA patients showed significantly higher methylation levels in cg11003133_79 compared to the HC group and the AS group non-responsive to anti-TNF-α treatment (*FDR* = 0.04 and 0.03). These findings imply that *AIM2* methylation patterns may serve as biomarkers for RA subtype stratification and treatment response (Figs. **1D**-**E**).

Furthermore, the association of *AIM2* methylation levels with clinical biomarkers and demographic characteristics was examined to evaluate its potential role in disease monitoring. RA patients with high CRP levels exhibited significantly increased methylation at cg11003133_79, cg11003133_91, and cg11003133_139 (*P* < 0.05), suggesting a possible link between inflammation and * AIM2* methylation. Additionally, older RA patients (aged > 60.46) demonstrated significantly lower methylation levels of cg11003133_79, cg11003133_91, and cg11003133_152 (*P* < 0.05), suggesting age-related methylation patterns. These findings highlight the potential of *AIM2* methylation as a clinically relevant marker for monitoring RA progression and activity (Figs. **1F**-**G**).

### 3.2 Correlation Between Methylation Levels of *AIM2* and Common Clinical Indices in RA Patients

To further explore the clinical significance of *AIM2* methylation, correlations were investigated between methylation levels at individual CpG sites and commonly used clinical indices in RA patients. These indices were selected based on their relevance to disease activity, inflammatory status, and comorbidities associated with RA, to understand how *AIM2* methylation may reflect clinical characteristics and disease progression. These indices included ESR, CRP, Rheumatoid Factor (RF), Cyclic Citrullinated Peptide (CCP), Disease Activity Score - 28 joints(DAS28)-ESR, DAS28-CRP, Visual Analogue Scale (VAS), number of tender joints, number of swollen joints, Disease Activity Index (CDAI), and presence of comorbidities such as hypertension, diabetes, and interstitial lung disease.

The analysis revealed significant correlations between individual CpG sites and specific clinical indices, which suggest potential clinical applications of *AIM2* methylation as disease-related biomarkers. For instance, cg11003133_79 is significantly positively correlated with ESR (ρ = 0.14, *q*=0.01), CRP (ρ = 0.12, *q* = 0.04), hypertension comorbidities (ρ = 0.20; *q* < 0.001), number of swollen joints (ρ = 0.16, *q* = 0.007), DAS28-ESR (ρ = 0.19, *q* < 0.001), DAS28-CRP (ρ = 0.15, *q* = 0.01), and CDAI, (ρ = 0.12, *q* = 0.044). cg11003133_91 significantly positively correlated with ESR (ρ = 0.17, *q* = 0.002), CRP (ρ = 0.13, *q* = 0.022), CCP (ρ = 0.12, *q* = 0.047), hypertension comorbidities (ρ = 0.15, *q* = 0.012), interstitial lung disease comorbidities (ρ = 0.18, *q* = 0.001), number of tender joints (ρ = 0.13, *q* = 0.022), number of swollen joints (ρ = 0.19, *q* = 0.001), DAS28-ESR (ρ = 0.23, *q* < 0.001), DAS28-CRP (ρ = 0.19, *q* = 0.001), and CDAI (ρ = 0.17, *q* = 0.002). cg11003133_139 is significantly positively correlated with ESR (ρ = 0.11, *P* = 0.033, q=0.060), CRP (ρ = 0.12, *q* = 0.046), hypertension comorbidities (ρ = 0.15, *q* = 0.008), DAS28-ESR (ρ =0.11, *P* = 0.028, *q* =0.054), and DAS28-CRP (ρ = 0.14, *q* = 0.018), cg11003133_152 is significantly positively correlated with CRP (ρ = 0.11, *P* = 0.029, *q* =0.054), and DAS28-CRP (ρ = 0.11, *P* = 0.042, *q* =0.074). Additionally, cg11003133_152 shows significant negative correlations with diabetes comorbidities (ρ = -0.12, *q* = 0.039) and the number of tender joints (ρ = -0.11, *P* = 0.041, *q* =0.073) (Fig. **S1**).

### 3.3 Significant Changes in *AIM2* Haplotype Methylation Levels Between RA and Other Diseases

**Fig. (2):**
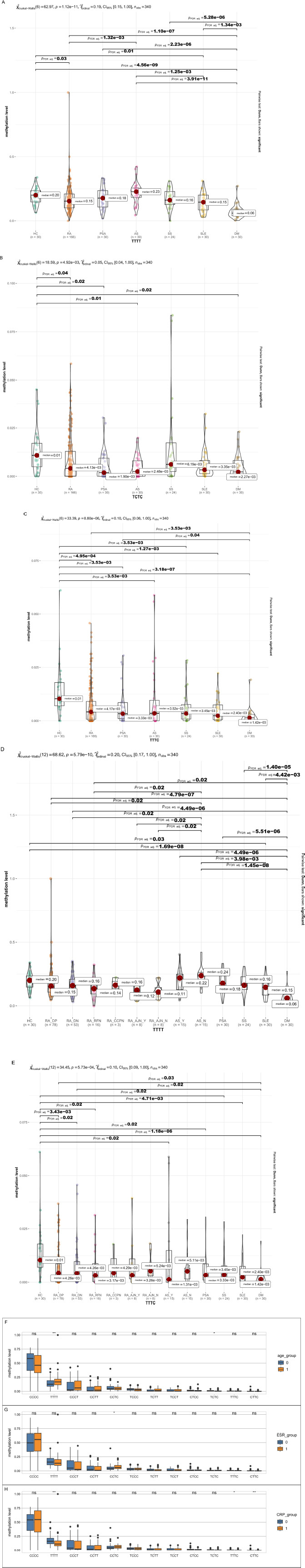
Haplotypic methylation levels of *AIM2* cg11003133. (**A-E**) Multiple comparisons of the haplotypic methylation levels of *AIM2* cg11003133 among multiple groups, with *FDR* <0.05 considered statistically significant. (**F-H**) Comparison of haplotypic methylation levels of *AIM2* cg11003133 in RA patients using different clinical cutoff values, with *P* <0.05 indicating statistical significance.

To investigate the broader methylation patterns in the *AIM2* region, 12 methylation haplotypes in the cg11003133 region were analyzed, and their differences between RA and other diseases were evaluated. This analysis aimed to determine whether haplotype-specific methylation variations could provide additional insights into the molecular distinctions of RA, its subtypes, and clinical characteristics, thus enhancing understanding of its epigenetic underpinnings. The findings revealed notable differences: the TTTT haplotype was significantly decreased in RA patients in both the HC (*FDR* = 0.03) and AS (*FDR* = 1.32 x 10^^-3^) groups. Conversely, the TTTT haplotype was significantly increased in RA patients (*FDR* = 1.10 x 10^^-7^) compared to the DM group. The TCTC and TTTC haplotypes were significantly decreased in RA patients compared to the HC group (*FDR* = 0.04 and 4.95 x 10^^-4^, respectively). Additionally, the TTTC haplotype was significantly increased in RA patients compared to the DM group (*FDR* = 3.01 × 10^-3^). These findings suggest that *AIM2* haplotype methylation changes may distinguish RA from other diseases and healthy controls (Figs. **2A**-**C**).

Further stratification of RA subtypes revealed additional insights. The TTTT haplotype was significantly reduced in RA patients with both positive RF/CCP, negative RF/CCP, single-negative RF, and in those regardless of their response to anti-TNF-alpha therapy (*FDR* = 0.02 for all) compared to the AS group unresponsive to anti-TNF-alpha therapy. Compared to the DM group, a significant increase in the TTTT haplotype was observed in RA patients with positive RF/CCP, negative RF/CCP, and single-negative RF (*FDR* = 4.79 x 10^^-7^, 4.49 x 10^^-6^, and 0.02, respectively). Compared to the HC group, the TTTC haplotype was notably decreased in RA patients with positive RF/CCP, negative RF/CCP, and single-negative RF (*FDR* = 3.43 x 10^^-3^, 0.02, and 0.02, respectively). In the DM group, a significant increase in the TTTC haplotype was observed in patients with RA, regardless of whether they had positive or negative RF/CCP ratios (*FDR* = 0.03 and 0.02, respectively). These subtype-specific patterns highlight the potential utility of haplotype methylation in distinguishing RA subtypes and their epigenetic profiles (Figs. **2D**-**E**).

The relationship between *AIM2* haplotype methylation and clinical cut-off values was examined to assess its relevance to disease activity and progression. RA patients with high CRP levels showed significant reductions in TTTT, TTTC, and CTTC haplotype methylation levels (*P* < 0.05), suggesting an association between inflammation and haplotype methylation. Moreover, in RA patients older than 60.46 years, the TTTT haplotype methylation level was significantly increased, while the TCTC haplotype methylation level was significantly decreased (*P* < 0.05) (Figs. **2F**-**H**). These findings suggest that specific haplotypes may be differentially influenced by age and inflammatory status, potentially serving as epigenetic markers for disease monitoring in RA.

### 3.4 Correlation of *AIM2* Haplotype Methylation Ratio with Common Clinical Indices in RA Patients

To explore the relationship between *AIM2* haplotype methylation and systemic inflammation in RA, correlations with key inflammatory markers (including CRP and ESR) were analyzed. The results revealed that the CCCC haplotype was significantly positively correlated with CRP levels (ρ = 0.14, *q* =0.023), suggesting its potential role in promoting inflammatory processes. Conversely, the TTTT haplotype showed a significant negative correlation with both CRP and ESR (ρ = -0.15 and -0.18; *q* = 0.012 and < 0.001, respectively), indicating that this haplotype may reflect a reduced inflammatory state. Similarly, the CTTC haplotype was negatively correlated with CRP levels (ρ = -0.10, *P* = 0.046, *q* =0.123). These findings suggest that specific haplotypes may serve as epigenetic markers of the inflammatory burden in RA (Fig. **S2**).

To assess the relevance of *AIM2* haplotype methylation to overall RA disease activity and joint involvement, correlations with composite disease activity scores (*e.g.*, DAS28-ESR and DAS28-CRP) and joint-specific parameters were examined. The CCTC haplotype was significantly positively correlated with DAS28-ESR and DAS28-CRP scores (ρ = 0.14 and 0.10; *P* = 0.007 and 0.050, *q* =0.025 and 0.130, respectively), as well as with the number of swollen joints (ρ = 0.10, *P* = 0.050, *q* =0.130). The TCCC haplotype was positively correlated with the number of tender joints (ρ = 0.11, *P* = 0.033, *q* =0.093), while the TCCT haplotype was negatively correlated with both the number of tender joints and CDAI (ρ = -0.14 and -0.11; *P* = 0.006 and 0.035, *q* =0.023 and 0.097, respectively). These results highlight the role of *AIM2* haplotype methylation in capturing both global and joint-specific disease activity in RA (Fig. **S2**).

A measure of pain perception was analyzed to understand the relationship between haplotype methylation and patient-reported outcomes, as well as correlations with the Visual Analog Scale (VAS) score. The CTCC haplotype was significantly negatively correlated with the VAS score (ρ = -0.15, *q* = 0.020), suggesting that this haplotype may be associated with reduced patient-reported pain severity. Additionally, the TCCT haplotype was also negatively correlated with the VAS score (ρ= -0.12, *P* = 0.022, *q* = 0.557). These findings provide insight into how *AIM2* methylation may influence subjective measures of disease burden and pain perception in RA patients (Fig. **S2**).

To investigate the broader clinical implications of *AIM2* haplotype methylation, associations with common RA comorbidities (including diabetes, hypertension, and interstitial lung disease) were analyzed. The CCCT and CCTT haplotypes showed significant positive correlations with diabetes (ρ = 0.17 and 0.16; *P* = 0.001 and 0.002, *q* =0.004 and 0.008, respectively), while the TTTT and TTTC haplotypes were negatively correlated with diabetes (ρ = -0.14 and -0.12; *P* = 0.008 and 0.019, *q* =0.028 and 0.060, respectively). For hypertension, the CCTT and TCTC haplotypes exhibited significant negative correlations (ρ = -0.13 and -0.11; *P* = 0.011 and 0.042, *q* =0.037 and 0.013, respectively). Interstitial lung disease was significantly positively correlated with the TCTC and TTTC haplotypes (ρ = 0.23 and 0.13; *q* < 0.001 and 0.037, respectively) but negatively correlated with the TCTT haplotype (ρ = -0.13, *P* = 0.043). These findings underscore the potential role of *AIM2* haplotype methylation in reflecting systemic disease manifestations and comorbidities in RA patients, highlighting its potential as a marker for predicting extra-articular complications (Fig. **S2**).

### 3.5 Methylation Levels of *AIM2* can Assist in RA Diagnosis

**Fig. (3):**
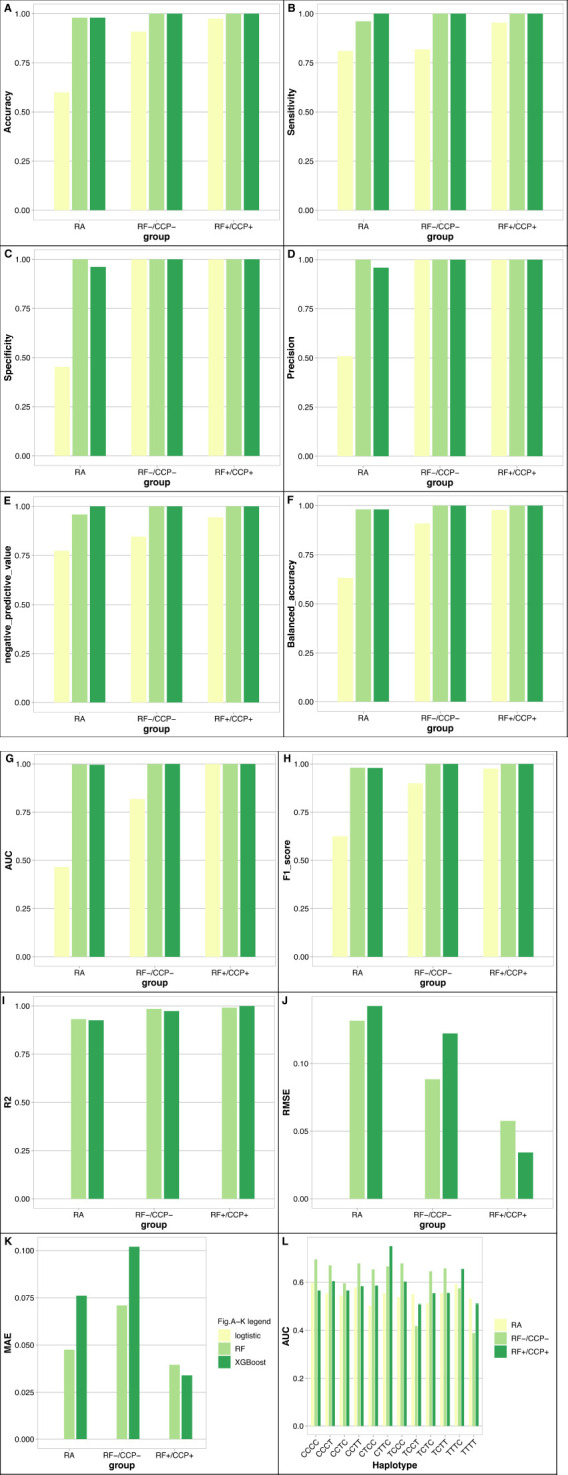
Clinical model test set results. (**A-K**) The performance of three different random forest methods in constructing models using different loci and average methylation of *AIM2* cg11003133 was evaluated on the test set. (**L**) Logistic regression was employed to assess the performance of different haplotype methylation levels of *AIM2* cg11003133 on the test set.

Diagnostic models incorporating logistic regression, XGBoost, and Random Forest algorithms were developed to evaluate the potential of *AIM2* cg11003133 as a biomarker for RA. This approach was chosen to ensure a comprehensive evaluation of model performance across diverse algorithms, as each method has unique strengths and assumptions that may influence predictive accuracy. The results indicated that modelling with four units of *AIM2* cg11003133 and average methylation levels significantly distinguished between RA and non-RA patients. Specifically, the model constructed using XGBoost demonstrated a validation set with an area under the curve (AUC) of 0.9911, an R-squared value (R2) of 0.8531, a root mean square error (RMSE) of 0.1927, a mean absolute error (MAE) of 0.0927, and an F1 score of 0.9545. The Random Forest model attained an AUC of 0.9975, an R2 of 0.9357, an RMSE of 0.1268, an MAE of 0.032, and an F1 score of 0.98 on the validation set. The Logistic model demonstrated an AUC of 0.4911 on the validation set, with an accuracy of 0.5556 (95% CI: 0.447-0.6604) and an F1 score of 0.5744 (Figs. **3A**-**K** and Table **S2**).

Further exploration of the potential of *AIM2* cg11003133 as a diagnostic biomarker for different RA subtypes revealed significant differentiation between RF/CCP double-negative and double-positive RA patients through combined modelling of the four units and average methylation levels of *AIM2* cg11003133. For patients with RF/CCP double-negative RA, the XGBoost model achieved a perfect AUC of 1, an R2 of 0.9959, an RMSE of 0.0877, an MAE of 0.0836, and an F1 score of 1 on the validation set. The RF model also achieved a perfect AUC of 1, an R2 of 0.9670, an RMSE of 0.1168, an MAE of 0.0883, and an F1 score of 1 in the validation set. The Logistic model showed an AUC of 0.8512 for the validation set, with an accuracy of 0.9091 (95% CI: 0.7084-0.9888) and an F1 score of 1. For RF/CCP double-positive RA patients, the XGBoost model achieved a perfect AUC of 1, an R2 of 0.9999, an RMSE of 0.0339, an MAE of 0.0339, and an F1 score of 1 on the validation set. The RF model also achieved a perfect AUC of 1, an R2 of 0.9673, an RMSE of 0.1116, an MAE of 0.0808, and an F1 score of 1 in the validation set. The Logistic model demonstrated a perfect AUC of 1, with an accuracy of 1 (95% CI: 0.9097-1) and an F1 score of 1 (Figs. **3A**-**K** and Table **S2**).

The diagnostic potential of *AIM2* haplotype methylation levels for RA was further evaluated using logistic regression analysis. The results revealed that *AIM2* haplotype methylation levels differed between RA and non-RA patients, with an AUC range of 0.463-0.5467; between double-negative and non-double-negative RA patients, with an AUC range of 0.4174-0.6942; and between double-positive and non-double-positive RA patients, with an AUC range of 0.509-0.7494 (Fig. **3L** and Table **S3**).

## 4 DISCUSSION

The role of *AIM2* in RA pathogenesis is increasingly recognized through its dual mechanisms of inflammasome-mediated pyroptosis and PANoptosis, both of which contribute to chronic synovial inflammation [4]. *AIM2*-driven pyroptosis involves the assembly of the *AIM2*-ASC-caspase-1 inflammasome complex, which cleaves pro-IL-1β and pro-IL-18 into their active forms, amplifying inflammatory cascades in RA synovium [4, 24]. Elevated serum *AIM2* levels and upregulated *AIM2*, ASC, caspase-1, and IL-1β expression in RA synovial tissues further underscore its pro-inflammatory role [25], with these biomarkers showing strong correlations with ESR and CRP levels—clinical hallmarks of systemic inflammation [26]. The observed hyperactivation of *AIM2* inflammasome pathways aligns with broader evidence that cytoplasmic dsDNA accumulation in RA synovial cells (from micronuclei, mitochondrial DNA leakage, or NETosis) serves as a persistent trigger for *AIM2* sensing, perpetuating a feedforward loop of cytokine release and tissue damage [27-31]. This study assessed DNA methylation changes at *AIM2* cg11003133 in peripheral blood mononuclear cells from RA patients and seven comparator diseases (including AS) using targeted bisulfite sequencing. Four CpG sites within the *AIM2* region (*chr1:159076528-159076740*) were analyzed, with cg11003133_79, cg11003133_91, and cg11003133_139 demonstrating significant methylation alterations in RA patients and subtypes compared to control groups. These differential methylation patterns showed potential associations with therapeutic response outcomes. The sites cg11003133_79, cg11003133_91, and cg11003133_139 are located within the 5-UTR and intronic regions. Generally, DNA methylation in different regions can affect gene expression, potentially leading to aberrant translation and post-translational regulation, and ultimately influencing disease development. However, the relationship between DNA methylation and gene expression requires further investigation. In addition, DNA methylation may exert its effects by modulating the gene expression of *AIM2*. Furthermore, methylation levels at both individual and average sites of *AIM2* cg11003133 were analyzed for correlations with common clinical indicators in RA patients. The positive correlations between cg11003133 methylation levels and inflammatory markers (ESR, CRP, DAS28) suggest that *AIM2* methylation status may serve as a dynamic regulator of disease activity. However, the exact directional relationship (whether methylation drives inflammation or *vice versa*) remains to be elucidated.

Methylation haplotypes representing coordinated changes across the four CpG sites were subsequently analyzed. Twelve distinct haplotypes demonstrated significant variation across RA patients, RA subtypes, and control groups. Proportional alterations in these haplotypes suggested potential genome-wide implications for both DNA methylation patterns and *AIM2* gene expression regulation. Spearman correlation analysis revealed significant positive associations between the CCCC haplotype and serum CRP levels, suggesting that coordinated methylation at the *AIM2* cg11003133 locus may reflect enhanced inflammatory responses in RA. The TTTT haplotype was mainly negatively correlated with CRP and ESR. In contrast, the TTTC haplotype was significantly negatively correlated with ESR, suggesting that overall demethylation of *AIM2* cg11003133 could indicate a weaker inflammatory response.

Additionally, various haplotypes are associated with RA comorbidities, including diabetes, hypertension, and interstitial lung disease. RA has multiple comorbidities; for instance, various pro-inflammatory factors can increase the incidence of hypertension [32]. Interstitial lung disease is a serious complication of RA with a significantly increased mortality rate compared to RA patients without interstitial lung disease [33]. The incidence of Insulin Resistance (IR) is significantly increased in RA patients and is independently correlated with MMP-3 levels and DAS28-ESR scores [34]. There is a mutual connection between RA, insulin resistance, and diabetes. Increased insulin resistance may lead to the release of pro-inflammatory factors that induce systemic inflammation, and drugs used in RA treatment seem to improve glucose metabolism [35]. In summary, the overall methylation level of *AIM2* cg11003133 may be significant in the study of RA comorbidities.

The diagnostic difficulties in Seronegative Rheumatoid Arthritis (SnRA) primarily stem from the absence of classic serological markers, such as RF and ACPA. These patients constitute 15%-25% of all RA cases [36], and their diagnosis heavily relies on clinical manifestations and the exclusion of other diseases. The lack of specific antibodies complicates early diagnosis, potentially leading to delayed treatment and an increased risk of joint destruction and disability [37-39]. For instance, some patients may initially present with asymmetric arthritis or atypical extra-articular symptoms, which can be easily confused with other inflammatory arthritides (*e.g.*, psoriatic arthritis, spondyloarthritis) or infectious diseases [40-42]. Imaging techniques partially compensate for the limitations of serological markers. Musculoskeletal ultrasound can detect early synovial hyperplasia and bone erosions, with studies demonstrating a correlation between ultrasound-detected synovitis and disease activity in ACPA-negative RA patients [43]. Furthermore, novel imaging modalities such as ^68^Ga-FAPI PET/CT, which targets fibroblast activation protein, enable the visualisation of synovial inflammation, providing objective biological evidence for SnRA [37]. However, the nonspecific nature of imaging findings may still lead to misdiagnosis, particularly in differentiating SnRA from other seronegative arthritides (*e.g.*, reactive arthritis or crystal-induced arthritis) [44, 45]. Recent research has focused on identifying alternative biomarkers for SnRA. Lipidomics studies have revealed distinct lipid metabolite profiles in the serum and urine of SnRA patients, which may serve as potential diagnostic indicators [46]. Additionally, novel autoantibodies such as anti-PTX3 and anti-PCOLCE antibodies show diagnostic potential for SnRA [47, 48]. Nevertheless, the sensitivity and specificity of these markers require further validation in large-scale studies [49, 50]. Notably, some SnRA patients may harbor *IDH* gene mutations, which are associated with innate immune dysregulation and alterations in the inflammatory microenvironment, suggesting potential pathophysiological differences from seropositive RA [51]. The diagnosis of SnRA necessitates a comprehensive evaluation integrating multiple parameters. Machine learning models incorporating clinical features (*e.g.*, joint swelling counts, morning stiffness duration) and laboratory markers (*e.g.*, CRP, ESR) have improved the accuracy of predicting progression from seronegative Undifferentiated Arthritis (UA) to RA [52]. Synovial biopsy remains valuable in challenging cases, aiding in the differentiation of rare conditions (*e.g.*, IgG4-related disease or Whipple’s disease) and preventing misdiagnosis [40, 41, 53]. Therapeutic response may also serve as a diagnostic clue, as favorable responses to JAK inhibitors (*e.g.*, upadacitinib) or glucocorticoid therapy may support an RA diagnosis [47, 54, 55]. In summary, the diagnosis of SnRA requires a comprehensive evaluation incorporating clinical manifestations, dynamic imaging findings, novel biomarkers, and treatment responses while excluding other potential diseases. Future research should focus on elucidating its distinct immunopathological mechanisms and developing more precise diagnostic tools to improve clinical management.

In the development of diagnostic tools for SnRA, peripheral blood samples offer significant advantages due to their accessibility, standardized collection procedures, and ability to reflect systemic immune status. Peripheral blood can be obtained non-invasively and repeatedly, making it suitable for dynamic monitoring of disease progression and treatment response, particularly in early diagnosis [46, 56, 57]. Studies indicate that immune cell dysregulation (*e.g.*, monocytes, T cells, B cells, and natural killer cells) in peripheral blood is closely linked to SnRA pathogenesis. Advanced techniques such as flow cytometry and single-cell RNA sequencing can identify disease-specific immune phenotypes, providing molecular-level diagnostic insights [56, 58-60]. Moreover, metabolomic and lipidomic analyses of peripheral blood have identified SnRA-specific metabolic signatures (*e.g.*, abnormal lipid profiles), which may serve as supplementary diagnostic markers [57, 61-63]. DNA methylation profiling and the detection of long non-coding RNA (lncRNA) in peripheral blood also demonstrate high diagnostic value [64]. For example, targeted sequencing of DNA methylation sites can effectively distinguish SnRA patients from healthy control groups [57, 62]. Compared to invasive samples such as synovial tissue, peripheral blood is more practical for large-scale clinical validation and translational applications, particularly in the development of multi-omics-based diagnostic models. The development of diagnostic approaches for SnRA patients has been significantly advanced by targeted DNA methylation studies in peripheral blood combined with machine learning algorithms. Targeted DNA methylation profiling enables precise detection of RA-related epigenetic alterations in peripheral blood. For instance, research using targeted sequencing revealed abnormal methylation levels at the *HIPK3* gene locus (cg15692052) in RA patients, demonstrating near-perfect diagnostic accuracy (approaching 100%) and outperforming conventional antibody-based tests in distinguishing seronegative RA [16, 57, 65]. Machine learning algorithms (*e.g.*, random forest, SVM-RFE) have further refined diagnostic precision by identifying an optimal panel of 81 methylation sites that can accurately classify seronegative RA patients *versus* healthy controls, even without relying on clinical parameters [66, 67]. This combinatorial biomarker approach exhibits superior diagnostic performance compared to single biomarkers and may potentially replace existing serological tests. The diagnosis of seronegative RA is particularly challenging due to the absence of specific antibodies (*e.g.*, RF and ACPA), forcing reliance on subjective clinical scoring and imaging findings, which often lead to misdiagnosis or delayed diagnosis. In contrast, peripheral blood DNA methylation markers directly reflect disease-associated epigenetic reprogramming in immune cells. For example, *CXCR5* gene methylation correlates significantly with RA disease activity (DAS28 score) independently of serological status [16, 68]. Fibroblast-Like Synoviocyte (FLS)- and monocyte-specific methylation signatures can be detected in peripheral blood, revealing early immune dysregulation in RA [69-71]. Machine learning algorithms integrate nonlinear relationships among multiple methylation sites to construct a “methylation fingerprint” capable of precise prediction, even for complex phenotypes such as the progression from undifferentiated arthritis to RA [52, 57, 72]. Peripheral blood sampling offers noninvasive collection, making it ideal for large-scale screening and frequent monitoring (*e.g.*, outpatient follow-up). Targeted methylation sequencing (*e.g.*, MethylTarget technology) requires minimal DNA input and reduces costs by over 90% compared to whole-genome sequencing [66]. While genetic risk loci (*e.g.*, *HLA-DRB1*) show limited predictive value for seronegative patients, methylation markers capture gene-environment interactions (*e.g.*, smoking-associated locus cg06690548), overcoming this limitation. Twin studies confirm that Differentially Methylated Regions (DMRs) in RA patients' peripheral blood are disease-associated, independent of genetic background [7, 66, 73]. Machine learning prioritizes methylation-driven sites linked to RA pathogenesis (*e.g.*, NF-κB pathway genes), thereby minimising interference from genetic heterogeneity [74, 75]. The integration of targeted DNA methylation analysis with machine learning algorithms has revolutionised the diagnostic paradigm for seronegative Rheumatoid Arthritis (RA), establishing a closed-loop system that encompasses high-precision biomarker screening, dynamic pathological tracking, and automated analysis. This innovative approach fundamentally transforms the traditional reliance on clinical phenotypes and serum antibodies, offering distinct advantages including antibody-independent diagnosis, genetic background-insensitive assessment, real-time disease monitoring, integrated treatment response prediction, and near-perfect classification performance. Collectively, this breakthrough represents a new gold standard for the diagnosis and management of RA. The study employed machine learning algorithms, including k-fold cross-validation and propensity score matching, to systematically evaluate the diagnostic utility of *AIM2* cg11003133 methylation. Results demonstrate the outstanding discriminatory power of this methylation signature. Notably, its diagnostic performance remains robust not only in seropositive (RF+/CCP+) subgroups but also in seronegative (RF-/CCP-) cases, providing a novel solution for the clinically challenging seronegative RA diagnosis.

This study has flaws that should be addressed in future studies. Firstly, the relationship between DNA methylation of *AIM2* cg11003133 and gene expression requires further investigation. The gene function study of *AIM2* in RA is one of the key topics our research group is focusing on, integrating cell biology, molecular biology, bioinformatics, and epigenetics to conduct a comprehensive and in-depth study of *AIM2*'s function, with valuable results anticipated. Secondly, these findings generally showed a trend towards weak correlation, necessitating validation with a larger sample size. However, the statistical support obtained was undeniable and provided insightful results. Thirdly, while statistically significant, most observed correlations exhibited weak effect sizes (|ρ| < 0.30), explaining less than 9% of variance (ρ^2^ < 0.09) in clinical indicators. This aligns with known challenges in epigenetic biomarker research, where methylation-gene expression relationships often show modest effect magnitudes due to biological complexity and technical noise. Although weak correlations may still hold clinical utility as composite biomarkers, their individual predictive power requires cautious interpretation and validation through multi-omics integration in larger cohorts. Finally, this study had certain limitations regarding the collection of sample information. The potential impact of medication regimens and disease duration on DNA methylation was not adequately considered. These factors may have significantly influenced the outcomes of this study. Firstly, different therapeutic approaches may significantly affect the DNA methylation patterns through various mechanisms. Medications can influence gene expression and epigenetic modifications, thereby altering DNA methylation. The potential variability introduced by medication may have been overlooked, which could affect the accuracy and interpretability of our results. Secondly, disease duration was a critical factor. Epigenetic characteristics may undergo dynamic changes as disease progresses. The lack of a detailed recording of disease duration may lead to an incomplete understanding of DNA methylation changes. Notably, the DNA methylation patterns of patients with long-term disease course could differ significantly from those of newly diagnosed patients. This aspect is not fully reflected in this study. These limitations impose certain constraints on the interpretation of the results. To address these shortcomings, sample collection and analysis methods should be improved in future studies by considering medication regimens and disease duration more comprehensively at the design stage, and meticulously recording and analysing these variables to ensure the precision of data collection and the reliability of the study conclusions. Additionally, the sample in this study exhibited a certain specificity in the age of onset, with higher onset ages for RA and gout and relatively lower onset ages for AS and SLE. Our sample selection reflects the actual age distribution of these diseases in the population, thereby enhancing the clinical relevance of our study results. In real-world clinical settings, patients with different diseases typically have different age distributions. By selecting representative clinical samples, our study more accurately reflects real-world scenarios and provides valuable insights into disease management across various age groups. Further refinement of the age matching could have enhanced the precision of our study. In future research, the aim is to expand the sample size and refine sample selection and matching, such as through propensity score matching, to reduce the impact of potential confounding factors on the study results. While the current sample size was empirically determined based on prior studies in the field, future work will incorporate formal power calculations to ensure robust detection of methylation effect sizes, particularly for subgroup analyses. Conducting larger-scale longitudinal studies will also provide deeper insights into the dynamic processes and time-dependent characteristics of DNA methylation changes. In summary, significant changes in the overall methylation levels of *AIM2* cg11003133 were identified in RA and its correlation with various clinical indicators, demonstrating its potential clinical utility as a novel diagnostic biomarker for RA, RA subtypes, and related complications.

## CONCLUSION

This study highlights the critical role of *AIM2* cg11003133 methylation in RA pathogenesis, particularly its association with inflammatory markers (ESR, CRP, and DAS28) and disease subtypes, including seronegative RA (SnRA). The differential methylation patterns at specific CpG sites (cg11003133_79, cg11003133_91, cg11003133_139) and distinct methylation haplotypes (*e.g.*, CCCC, TTTT) suggest that *AIM2* epigenetic regulation contributes to RA progression and comorbidities (*e.g.*, diabetes, interstitial lung disease). Furthermore, targeted DNA methylation profiling in peripheral blood, combined with machine learning, demonstrates high diagnostic accuracy for SnRA, offering a promising antibody-independent biomarker to overcome current diagnostic challenges. However, the weak correlation effect sizes and unresolved mechanistic links between methylation and *AIM2* expression warrant further investigation. Future studies should expand sample sizes, incorporate longitudinal designs, and integrate multi-omics approaches to validate these findings and explore therapeutic implications. Overall, this work advances the understanding of epigenetic dysregulation in RA and paves the way for precision diagnostics and personalized treatment strategies.

## Figures and Tables

**Table 1:** Basic clinical information.

Variables	Total (n=370)	HC (n=30)	RA (n=166)	AS (n = 30)	GOUT (n = 30)	PSA (n = 30)	SS (n = 24)	SLE (n = 30)	DM (n = 30)	Statistic	*P*
Gender, n(%) Male Female	128 (34.59) 242 (65.41)	15 (50.00) 15 (50.00)	25(15.06) 141 (84.94)	25 (83.33) 5 (16.67)	26 (86.67) 4 (13.33)	24 (80.00) 6 (20.00)	1 (4.17) 23 (95.83)	2 (6.67) 28 (93.33)	10 (33.33) 20 (66.67)	χ^2^=145.72	<0.0001
Age, M (Q_1_, Q_3_)	58.00(47.00, 66.00)	47.50(41.00, 52.75)	61.00(63.00, 69.00)	39.50(28.50, 52.75.00)	61.00(40.75, 76.75)	57.00(46.00, 66.00)	60.50(53.00, 65.00)	47.50(37.25, 59.25)	59.00(50.25, 68.25)	χ^2^=69.44	<0.0001
Height, M (Q_1_, Q_3_)	162.00(158.00, 170.00)	162.50(158.00, 170.00)	160.00(155.00, 165.00)	171.50(167.50, 177.00)	170.00(160.00, 175.00)	168.00(164.00, 171.00)	168.00(164.00, 171.00)	163.00(160.00, 164.00)	168.00(164.00, 171.00)	χ^2^=68.02	<0.0001
Weight, M (Q_1_, Q_3_)	60.00 (54.00, 70.00)	64.00(54.00, 69.00)	57.00(50.25, 65.00)	75.00(65.00, 85.00)	69.00(63.50, 80.00)	66.50(60.00, 76.25)	54.00(51.00, 61.50)	62.50(55.70, 73.50)	57.00(55.00, 64.00)	χ^2^=61.82	<0.0001
ESR, M (Q_1_, Q_3_)	15.00 (7.00, 29.00)	-	10.00 (20.00, 31.00)	5.50 (3.75, 12.25)	9.00 (4.00, 21.50)	7.50 (4.00, 15.50)	17.50 (7.00, 30.00)	47.00 (15.25, 75.75)	20.50 (9.00, 29.25)	χ^2^=59.96	<0.0001
CRP, M (Q_1_, Q_3_)	1.23 (0.50, 7.62)	-	1.39 (0.50, 11.40)	1.31 (0.50, 6.46)	1.51 (0.50, 6.52)	1.31 (0.50, 3.80)	0.54 (0.50, 3.30)	1.04 (0.50, 8.51)	0.50 (0.50, 3.45)	χ^2^=7.81	0.253
RF, M (Q_1_, Q_3_)	-	-	21.00 (9.19, 108.73)	-	-	-	-	-	-	-	-
CCP, M (Q_1_, Q_3_)	-	-	207.40 (20.00, 843.98)	-	-	-	-	-	-	-	-
Tender joints, M (Q_1_, Q_3_)	-	-	2.00(0.00, 4.00)	-	-	-	-	-	-	-	-
Swollen joints, M (Q_1_, Q_3_)	-	-	1.00(0.00, 2.00)	-	-	-	-	-	-	-	-
VAS, M (Q_1_, Q_3_)	-	-	30.00(20.00, 50.00)	-	-	-	-	-	-	-	-
DAS28-ESR, M (Q_1_, Q_3_)	-	-	3.43(2.55, 4.21)	-	-	-	-	-	-	-	-
DAS28-CRP, M (Q_1_, Q_3_)	-	-	3.75(3.05, 4.63)	-	-	-	-	-	-	-	-
CDAI, M (Q_1_, Q_3_)	-	-	9.00(6.75, 14.00)	-	-	-	-	-	-	-	-

**Abbreviations:** RA, rheumatoid arthritis; AS, ankylosing spondylitis; PsA, psoriatic arthritis; SLE, systemic lupus erythematosus; DM, dermatomyositis; SS, primary Sjögren's syndrome; HC, healthy controls.

## Data Availability

The datasets presented in this study are available in online repositories. The names of the repository/repositories and accession number(s) can be found below: SRA, PRJNA1094652.

## LIST OF ABBREVIATIONS

RA: Rheumatoid Arthritis
RF: Rheumatoid Factor
CCP: Cyclic Citrullinated Peptide
PRR: Pattern Recognition Receptor
MHC: Major Histocompatibility Complex
FLS: Fibroblast-like Synoviocytes
PTEN: Phosphatase and Tensin Homologue
IFNs: Interferons
AS: Ankylosing Spondylitis
PsA: Psoriatic Arthritis
SLE: Systemic Lupus Erythematosus
DM: Dermatomyositis
HC: Healthy Controls
SS: Sjögren’s Syndrome
VAS: Visual Analogue Scale

## STANDARDS OF REPORTING

STROBE guidelines were followed.
