# Supplementary material for: Identifying AIM2 Circulating Methylation Levels as a Novel Diagnostic Biomarker for Rheumatoid Arthritis Using Targeted DNA Methylation Sequencing
Source: Endocr Metab Immune Disord Drug Targets. 2025 Jul 17;26:E18715303401357. doi: 10.2174/0118715303401357250707080740 (PMC13334255; doi:10.2174/0118715303401357250707080740)
Supplement: Supplementary file 1 [file EMIDDT-26-E18715303401357_SD1.pdf]

Supplementary Material

Identifying AIM2 Circulating Methylation Levels as a Novel Diagnostic Biomarker for Rheumatoid Arthritis Using Targeted DNA Methylation Sequencing

Jianan Zhao[1,2,3,#], Binghen He[4,#], Yu Shan[1,2,3,#] , Kai Wei[1,2,3] , Ping Jiang[1,2,3] , Yiming Shi[1,2,3] , Cen Chang[1,2,3] , Yixin Zheng[1,2,3] , Fuyu Zhao[1,2,3] , Yunshen Li[1,2,3] , Yuejuan Zheng[5] , Yehua Jin[1,2,3,*] , Xinliang Lv[6,*] and Mengru Guo[1,2,3,*]

[1]Department of Rheumatology, Shanghai Guanghua Hospital of Integrative Medicine, Shanghai University of Traditional Chinese Medicine, Shanghai, China; [2]Guanghua Clinical Medical College, Shanghai University of Traditional Chinese Medicine, Shanghai, China; [3]Institute of Arthritis Research in Integrative Medicine, Shanghai Academy of Traditional Chinese Medicine, Shanghai, China; [4]Shanghai University of Traditional Chinese Medicine Affiliated Shuguang Hospital, Shanghai, China; [5]The Research Center for Traditional Chinese Medicine, Shanghai Institute of Infectious Diseases and Biosecurity, Shanghai University of Traditional Chinese Medicine, Shanghai, China; [6]Traditional Chinese Medicine Hospital of Inner Mongolia Autonomous Region, Hohhot, Inner Mongolia Autonomous Region, China

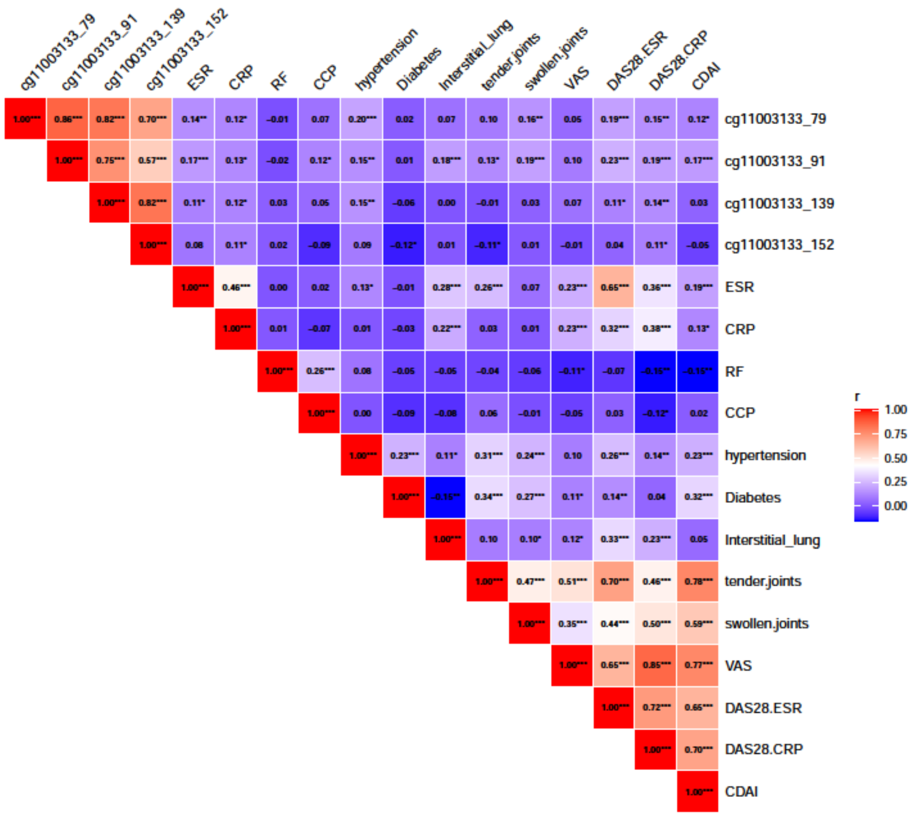

Fig. (S1) Correlation between Methylation Levels of *AIM2* and Common Clinical Indices in RA Patients. Correlation between *AIM2* Methylation Levels and Common Clinical Indices in RA Patients

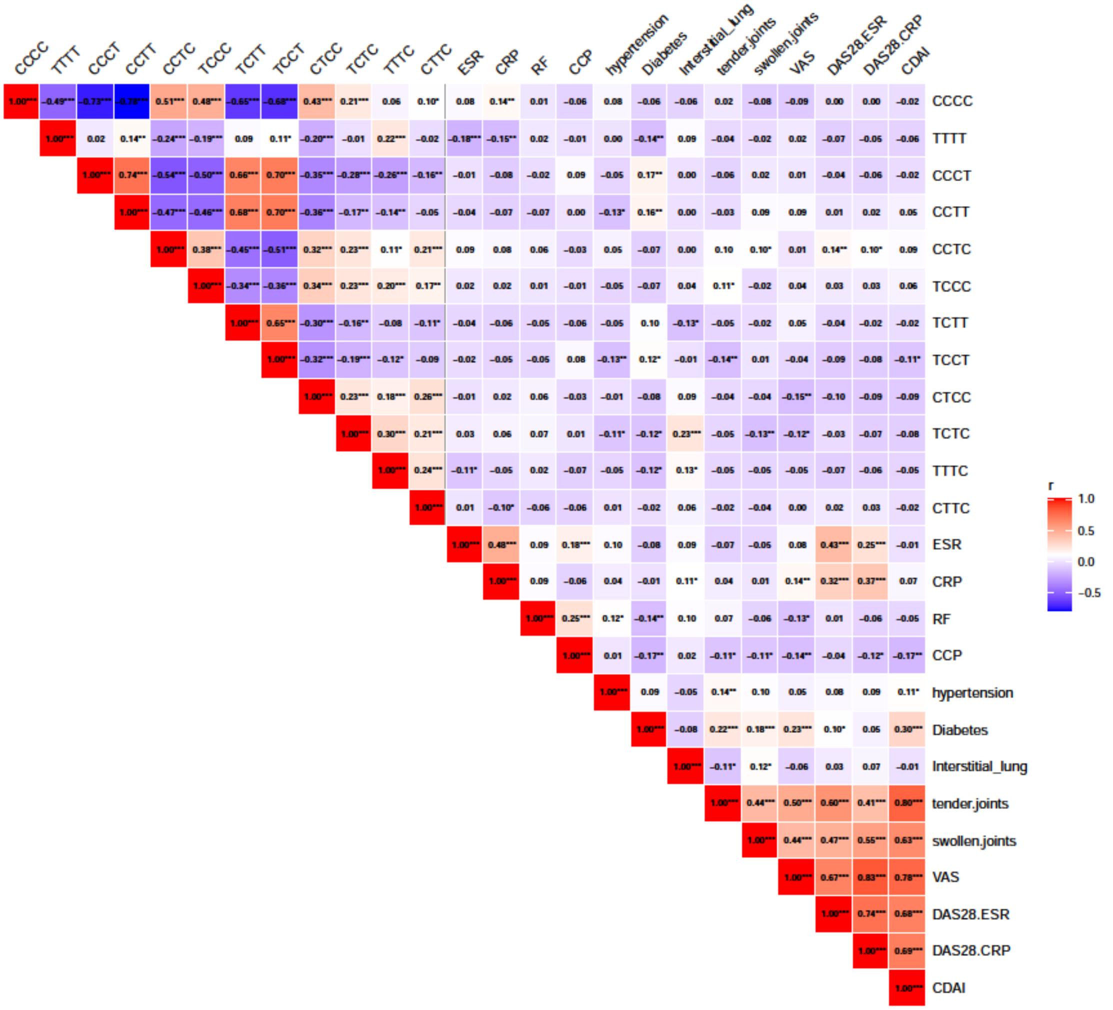

Fig. (S2) Correlation of *AIM2* Haplotype Methylation Levels with Common Clinical Indices in RA Patients. Correlation of *AIM2* Haplotype Methylation Levels with Common Clinical Indices in RA Patients.

Table S3：Clinical model test set results for haplotype

	Accuracy (95%CI)	Sensi-tivity	Speci-ficity	Precision	Negative Predictive Value	Balanced Ac-Curacy	AUC	Method
CCCC	0.3182(0.1386, 0.5487)	0.18182	0.45455	0.25	0.35714	0.31818	0.6942	RF-/CCP-
TTTT	0.5909(0.3635, 0.7929)	0.7273	0.4545	0.5714	0.625	0.5909	0.3884	RF-/CCP-
CCCT	0.3182(0.1386, 0.5487)	0.09091	0.54545	0.16667	0.375	0.31818	0.6694	RF-/CCP-

CCTT	0.3636(0.172, 0.5934)	0.09091	0.63636	0.2	0.41176	0.36364	0.6777	RF-/CCP-
CCTC	0.4545(0.2439, 0.6779)	0.4545	0.4545	0.4545	0.4545	0.4545	0.595	RF-/CCP-
TCCC	0.4545(0.2439, 0.6779)	0.4545	0.4545	0.4545	0.4545	0.4545	0.6777	RF-/CCP-
TCTT	0.4545(0.2439, 0.6779)	0.18182	0.72727	0.4	0.47059	0.45455	0.657	RF-/CCP-
TCCT	0.6364 (0.4066, 0.828)	0.9091	0.3636	0.5882	0.8	0.6364	0.4174	RF-/CCP-
CTCC	0.5(0.2822, 0.7178)	0.4545	0.5455	0.5	0.5	0.5	0.6529	RF-/CCP-
TCTC	0.3182(0.1386, 0.5487)	0.2727	0.3636	0.3	0.3333	0.3182	0.6446	RF-/CCP-
TTTC	0.5(0.2822, 0.7178)	0.7273	0.2727	0.5	0.5	0.5	0.5744	RF-/CCP-
CTTC	0.3636(0.172, 0.5934)	0.5455	0.1818	0.4	0.2857	0.3636	0.6653	RF-/CCP-
CCCC	0.475(0.3151, 0.6387)	0.3478	0.6471	0.5714	0.4231	0.4974	0.5652	RF+/CCP+
TTTT	0.425(0.2704, 0.5911)	0.3478	0.5294	0.5	0.375	0.4386	0.509	RF+/CCP+
CCCT	0.375(0.2273, 0.542)	0.3043	0.4706	0.4375	0.3333	0.3875	0.6036	RF+/CCP+
CCTT	0.525(0.3613, 0.6849)	0.3043	0.8235	0.7	0.4667	0.5639	0.5831	RF+/CCP+
CCTC	0.325(0.1857, 0.4913)	0.1739	0.5294	0.3333	0.3214	0.3517	0.5652	RF+/CCP+
TCCC	0.425(0.2704, 0.5911)	0	1	0	0.425	0.5	0.6023	RF+/CCP+
TCTT	0.425(0.2704, 0.5911)	0.2174	0.7059	0.5	0.4	0.4616	0.555	RF+/CCP+
TCCT	0.475(0.3151, 0.6387)	0.1304	0.9412	0.75	0.4444	0.5358	0.5077	RF+/CCP+
CTCC	0.4(0.2486, 0.5667)	0	0.9412	0	0.4103	0.4706	0.5857	RF+/CCP+
TCTC	0.5 (0.338, 0.662)	0.5217	0.4706	0.5714	0.4211	0.4962	0.5537	RF+/CCP+
TTTC	0.425(0.2704, 0.5911)	0	1	0	0.425	0.5	0.6547	RF+/CCP+
CTTC	0.425(0.2704, 0.5911)	0	1	0	0.425	0.5	0.7494	RF+/CCP+

CCCC	0.4301(0.3278, 0.5369)	1	0	0.4301	0	0.5	0.5972	RA
TTTT	0.4194(0.3178, 0.5262)	0.775	0.1509	0.4079	0.4706	0.463	0.5314	RA
CCCT	0.4301(0.3278, 0.5369)	1	0	0.4301	0	0.5	0.5531	RA
CCTT	0.4301(0.3278, 0.5369)	1	0	0.4301	0	0.5	0.5778	RA
CCTC	0.5054(0.3997, 0.6107)	0.85	0.2453	0.4595	0.6842	0.5476	0.5443	RA
TCCC	0.4301(0.3278, 0.5369)	1	0	0.4301	0	0.5	0.5377	RA
TCTT	0.4301(0.3278, 0.5369)	1	0	0.4301	0	0.5	0.5545	RA
TCCT	0.4301(0.3278, 0.5369)	1	0	0.4301	0	0.5	0.55	RA
CTCC	0.4301(0.3278, 0.5369)	1	0	0.4301	0	0.5	0.5021	RA
TCTC	0.4194 (0.3178, 0.5262)	0.975	0	0.4239	0	0.4875	0.5127	RA
TTTC	0.4301(0.3278, 0.5369)	0.85	0.1132	0.4198	0.5	0.4816	0.592	RA
CTTC	0.4301(0.3278, 0.5369)	1	0	0.4301	0	0.5	0.5538	RA

Note: RA, rheumatoid arthritis; RF-/CCP-, RF/CCP double negative RA patients; RF+/CCP+ , RF/CCP double positive RA patients
